# A guideline-based preference elicitation tool to enhance shared decision-making in supervised exercise therapy for patients with intermittent claudication: a process evaluation

**DOI:** 10.1080/07853890.2025.2540022

**Published:** 2025-08-04

**Authors:** Laura H. M. Marcellis, Katrien M. Rutgers, Steffie Spruijt, Joep A. W. Teijink, Philip J. van der Wees, Thomas J. Hoogeboom

**Affiliations:** ^a^ IQ Health science department, Radboud university medical center, Nijmegen, The Netherlands; ^b^ Chronisch ZorgNet, Eindhoven, The Netherlands; ^c^ Physique Preventiecentrum B.V, Arnhem, The Netherlands; ^d^ Department of Surgery, Catharina Hospital, Eindhoven, The Netherlands

**Keywords:** Peripheral arterial disease, intermittent claudication, physical therapy, implementation, shared decision-making, preference elicitation

## Abstract

**Background:**

To facilitate person-centered physical therapy for patients with intermittent claudication, we introduced a preference elicitation tool to Dutch physical therapists. The tool is designed to support the patient-therapist dialogue and enhance collaborative treatment planning. Successful integration of this tool into practice requires a thorough understanding of factors that influenced therapists’ (non)adoption.

**Objectives:**

To evaluate whether therapists adopted the preference elicitation tool, assess the tool’s reach, and identify perceived barriers and facilitators by therapists that influenced adoption.

**Methods:**

A multi-method process evaluation was conducted following a series of implementation activities. Routinely collected quantitative data were used to assess adoption and reach. Adoption was measured by the percentage of therapists who completed the tool’s e-learning module and the percentage of therapists who started using the tool. Reach was measured by the percentage of eligible patients with whom therapists actually used the tool. To identify barriers and facilitators, qualitative semi-structured interviews with eleven therapists were conducted and deductively analysed using the Tailored Implementation for Chronic Diseases framework.

**Results:**

Of the 1,130 therapists eligible to use the preference elicitation tool, 64% completed the e-learning. Among these, 45% started using the tool in clinical practice. Therapists used the tool with 38% of eligible patients. In total, 39 barriers and 37 facilitators for tool adoption were identified. Barriers included time investment, discomfort with the formal communication setting, and conflicts with activity-oriented role perceptions. Facilitators included the tool’s user-friendliness, flexible use of the tool, and enhanced patient engagement.

**Conclusion:**

Modest adoption and reach rates indicate opportunities for improving uptake and sustainable implementation of the preference elicitation tool. Therapists’ decisions to adopt the tool were influenced by various factors. Providing therapists with strategies to maintain a flexible approach to using the tool could address key barriers. Future research should explore patients’ perspectives on the preference elicitation tool.

## KEY MESSAGES

- Low to moderate levels of shared decision-making in current physical therapy practice requires the implementation of a preference elicitation tool to support patients in taking an active role in managing their long-term health and ultimately achieve optimal outcomes.
- This study evaluated the adoption and reach of a preference elicitation tool designed to enhance shared decision-making between physical therapists and patients with intermittent claudication as well as its barriers and facilitators for adoption.
- Addressing key barriers and leveraging facilitators, such as providing strategies to integrate the tool into therapists’ workflows and fostering reflective practice around shared decision-making, could improve adoption and reach and inform the development of tailored implementation strategies.

## Introduction

Intermittent claudication is a common symptom of peripheral arterial disease (PAD), causing cramping leg pain induced by physical exertion that limits patients’ ability to perform daily activities such as walking [1]. According to the stepped-care approach, supervised exercise therapy (SET) under guidance of a physical therapist is the first-choice treatment [2–4]. However, managing this chronic condition extends beyond following a prescribed exercise plan; it necessitates patients to make sustainable lifestyle changes that support long-term health [2]. This, in turn, depends on patients taking an active role in managing their health [5]. Achieving optimal outcomes therefore hinges on patients’ active involvement in their care decisions [6,7]. Shared decision-making is ideally suited to this context, as it fosters healthcare professionals and patients to jointly make treatment decisions that align with the patient’s needs, values and preferences [8–10].

Although shared decision-making has demonstrated significant benefits and is increasingly regarded standard practice, its implementation in clinical practice remains a challenge [11–13]. Decision aids have been proposed as a strategy to facilitate this process [14]. In line with this, we developed a preference elicitation tool for the physical therapy treatment of patients with intermittent claudication [15]. The tool is an encounter decision aid (i.e. meant for use within the consultation [16] designed to facilitate shared discussions of guideline-based treatment options and elicit patient preferences, thereby explicitly supporting the patient-therapist dialogue [17]. The preference elicitation tool builds on an earlier innovation, personalized outcomes forecasts (POFs), which provide a personalized prognosis of treatment outcomes [18]. In 2022, we introduced the preference elicitation tool to physical therapists as part of nationwide cluster randomized trial [15]. Results from this trial suggest that use of the preference elicitation tool is effective in enhancing shared decision-making and person-centered care [15]. This promising trend implies the potential value of achieving broad adoption and sustainable implementation in clinical practice.

Nevertheless, the implementation of innovations in healthcare can be complex, influenced by issues like innovation design, changes in workflow, and required time commitment [19–21]. Insights from our process evaluation of the POFs implementation emphasized that adoption by therapists is a multifaceted process influenced by various factors [22]. To ensure successful integration of the preference elicitation tool in clinical practice, understanding factors that influenced its implementation is essential. Therefore, we conducted a process evaluation of the preference elicitation tool’s implementation in the physical therapy treatment of patients with intermittent claudication. Our research objectives were to:

- 1) evaluate therapists’ adoption of the preference elicitation tool;
- 2) evaluate the reach of the preference elicitation tool among therapists who initiated its use, and;
- 3) identify barriers and facilitators that influenced adoption of the preference elicitation tool, as perceived by therapists.

## Methods

### Study design and setting

**Figure 1.**
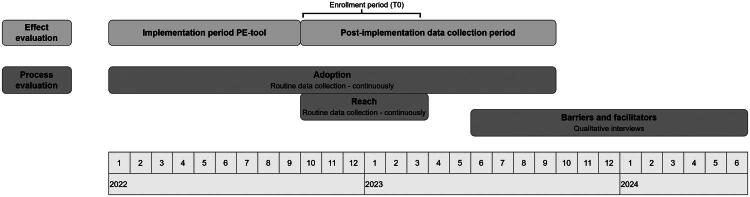
Visual representation of the study timeline. Abbreviations: PE-tool: preference elicitation tool.

This process evaluation was performed within the intervention arm of the previously conducted effect evaluation [15]. We conducted a multi-methods approach, with routinely collected quantitative data to evaluate adoption and reach, and qualitative semi-structured interviews to evaluate barriers and facilitators for using the preference elicitation tool (Figure 1). The study took place within the Dutch nationwide network called ‘Chronic CareNet’ [23], between January 2022 and August 2024 (ClinicalTrials.gov Identifier: NCT05232474). The Consolidated Criteria for Reporting Qualitative research (COREQ) checklist was used as reporting guideline [24].

### Implementation of the preference elicitation tool

We developed the preference elicitation tool for collaborative use between therapists and patients during the clinical encounter. The tool is designed to facilitate a shared discussion on setting-up a tailored treatment plan, specifically at the start of treatment and during periodic evaluations. Using the tool is meant to support therapists in actively engaging patients and elicit patients’ preferences on various decision topics, such as training modalities and lifestyle modifications. Treatment options presented in the tool are based on the Royal Dutch Society for Physical Therapy (KNGF) guideline on intermittent claudication [17]. The tool is online accessible through the web-based portal of Chronic CareNet. A visual impression of the tool is presented in Supplementary Figures 1–5.

We introduced the preference elicitation tool along with an accompanying e-learning module to physical therapists affiliated with Chronic CareNet who were randomized to the intervention arm of the cluster trial [15]. At the start of the nine-month implementation period, therapists received an email informing them of the free availability of the preference elicitation tool and e-learning. The e-learning, which took approximately one hour to complete, covered the basic usage and technical aspects of the tool. Completion of the e-learning was required before therapists could begin using the tool in their clinical practice. Use of the preference elicitation tool was not mandatory, which allowed therapists to voluntarily choose to use the tool or not. We conducted different implementation activities throughout the implementation period to encourage therapists to complete the e-learning and start or continue using the preference elicitation tool in practice (Table 1).

**Table 1.** Overview of the implementation activities during the implementation period.

Implementation activity	When (frequency)
Launch of the preference elicitation tool and supplementary e-learning.	Jan 2022 (1 time)
Invitations to therapists by email to complete the e-learning and start using the preference elicitation tool.	Jan 2022 (1 time)
Daily support for therapists with additional questions *via* email or phone.	Jan – Sep 2022 (continuous)
Reminder emails to therapists to complete the e-learning and start using the preference elicitation tool.	Feb – Sep 2022 (monthly)
Embedding the discussion of the use of the preference elicitation tool in multiple Chronic CareNet regional meetings.	Mar – Apr 2022 (continuous)
Organization of free (online) educational sessions for therapists on the use of the preference elicitation tool.	May – Jul 2022 (5 times)
Setting up a pop-up notification in the therapists’ Chronic CareNet dashboard encouraging them to complete the e-learning and start using the preference elicitation tool.	May – Sep 2022 (continuous)
Development and distribution of digital promotion material (e.g. video, infographic, notifications in Chronic CareNet newsletters).	May – Sep 2022 (monthly)

### Quantitative assessment (adoption and reach)

#### Participants

To evaluate adoption, data of physical therapists affiliated with Chronic CareNet, specialized in intermittent claudication treatment, and randomized to the intervention group of the cluster trial were included. To evaluate reach, data of patients referred for physical therapy treatment due to intermittent claudication were included after obtaining consent for the use of their clinical data *via* a digital signature.

#### Data collection

##### Adoption

Data on adoption were gathered from the start of the nine-month implementation period until the end of the subsequent twelve-month post-implementation period. We defined adoption as the *intention to try* and *action to try* the preference elicitation tool, according to Proctor et al. [25]

*Intention to try* represented the therapists who expressed interest in the preference elicitation tool. *Intention to try* was operationalized as a percentage (0–100%), with the numerator representing the number of therapists who completed the e-learning before the end of the post-implementation period. The denominator represented the total number of therapists eligible to use the tool at implementation start. *Intention to try* was evaluated using routinely collected administrative data from Chronic CareNet.

*Action to try* represented the therapists who actually used the preference elicitation tool in their clinical practice. *Action to try* was operationalized as a percentage (0–100%), with the numerator representing the number of therapists who used the tool at least once before the end of the post-implementation period, regardless the number of patients with intermittent claudication under treatment. The denominator represented the total number of therapists who completed the e-learning before the end of the post-implementation period. *Action to try* was evaluated using routinely collected data from the POFs database, which contains pseudonymized data of patients with intermittent claudication with whom therapists used the preference elicitation tool and/or the POFs.

##### Reach

Data on *reach* were gathered during the first six months of the post-implementation period. In the cluster trial, this same period constituted the enrollment phase, during which baseline measurements were routinely gathered for newly referred patients with intermittent claudication [15]. *Reach* was defined as the number of newly referred patients with intermittent claudication with whom the preference elicitation tool was used, relative to the total number of newly referred patients eligible for using the tool. *Reach* was operationalized as a percentage (0–100%), with the numerator representing the number of patients with intermittent claudication who started their physical therapy treatment during the six-month period and with whom the tool was used. The denominator represented the total number of patients with intermittent claudication who started their physical therapy treatment during the same period. We restricted both the numerator and denominator to patients under treatment by therapists who had taken *action to try* the preference elicitation tool. The numerator was estimated using routinely collected data from the POFs database, while the denominator was estimated using routinely collected data from the Chronic CareNet Quality system. The Quality system database contains pseudonymized data from electronic health records of patients with intermittent claudication who were referred to therapists affiliated with Chronic CareNet.

#### Data analysis

Descriptive statistics were used to show adoption and reach scores and to summarize demographic characteristics. Data were analysed using IBM SPSS Statistics v29.0. Categorical variables were reported as numbers with percentages, and continuous variables as means with standard deviations (SD) or medians with interquartile ranges (IQR), depending on the distribution of the data.

### Qualitative assessment (barriers and facilitators)

#### Participants

Therapists affiliated with Chronic CareNet and specialized in intermittent claudication treatment were eligible to participate if they were randomized to the intervention group of the cluster trial and completed the e-learning on the preference elicitation tool. We purposefully sampled therapists with varying levels of adoption of the preference elicitation tool (i.e. no adoption, low to moderate adoption, high adoption). No adoption meant therapists completed the e-learning but did not initiate use of the tool, low to moderate meant using the tool one to five times, and high adoption meant using the tool more than ten times.

#### Data collection

Therapists were invited to participate *via* email and/or telephone. When agreeing to participate, individual semi-structured interviews were conducted online using MS Teams. Interviews were conducted by two female members of the research team (LM and KR). Both are certified physical therapists with a master’s degree in clinical health sciences and have received training in qualitative research. At the time of the study, LM was employed as a PhD candidate and KR as a physical therapist. LM conducted interviews with therapists who demonstrated no adoption or low to medium adoption, while KR interviewed therapists with high adoption. This division allowed each interviewer to become familiar with the context of the specific subgroup, thereby facilitating more focused exploration. Neither LM nor KR knew therapists before conducting the interviews. Interviews took place between June 2023 and June 2024 and took ∼30 to 60 min. A semi-structured interview guide (Supplementary File 1) was developed by LM, KR and TH, based on the Tailored Implementation for Chronic Diseases (TICD) framework [26]. The TICD framework defines 57 potential determinants of practice relevant for the evaluation of implementation in healthcare practice. Determinants are grouped under seven main domains: 1) innovation factors, 2) individual health professional factors, 3) patient factors, 4) professional interactions, 5) incentives and recourses, 6) capacity for organizational change, and 7) social, political and legal factors. Both interviewers conducted a pilot interview to refine the interview guide and ensure clarity in asking questions. Prior to the interview, demographic information was collected using an online questionnaire in Castor EDC. Interviews were conducted until data saturation was reached, as determined by discussion between LM and KR on when no new information emerged [27]. Interviews were audio-recorded and transcribed verbatim by a third party.

#### Data analysis

We analyzed the data using framework analysis [26,28]. First, we read and re-read transcripts for familiarization with the data. Then, we identified relevant text fragments as a barrier or facilitator for implementation and coded these deductively using the 57 determinants from the TICD framework. We remained open for potential barriers and facilitators that did not align with determinants in the TICD framework. The first five transcripts were independently coded by KR and LM, after which the codes were compared and discussed to achieve consensus. For the remaining transcripts, coding was first performed by either KR or LM, subsequently reviewed by the other, and then codes were discussed by both to ensure agreement. Any discrepancies were discussed with a third researcher (TH). Final barriers and facilitators were refined by LM and subsequently discussed, refined, and agreed upon in a consensus meeting with KR, LM, PvdW, SS and TH. Data analysis was conducted using ATLAS.ti v23.1.1.

#### Trustworthiness

Credibility was enhanced through member checks, whereby interview transcripts were sent to participating therapists to ensure the accuracy of their interpretations. Transferability was addressed by providing detailed descriptions of the research context, methods, and participant characteristics. Confirmability and dependability were enhanced by taking fieldnotes and memos and by maintaining a comprehensive logbook during interview and analysis processes. Furthermore, KR and LM held regular meetings to evaluate the coding framework, explore alternative interpretations and deliberate on the clinical implications of the findings. Additionally, KR and LM used bracketing throughout the analysis process to set aside preconceptions.

### Ethical considerations

Full ethical consideration was waived by the Ethics Committee of Arnhem-Nijmegen in accordance with the Dutch Medical Research Involving Human Subjects Act (2021-13373). This study was conducted in accordance with the principles of the Declaration of Helsinki. All participating therapists agreed to the terms and conditions of Chronic CareNet, including compliance with data delivery procedures. The databases used only contained data from patients who provided consent *via* a digital signature for the use of their clinical data for research and clinical purposes [29]. Interviewed therapists were separately informed and asked for written consent before taking part. Participating therapists were reimbursed €50 in the form of a gift card.

## Results

### Quantitative assessment (adoption and reach)

#### Adoption

At the start of implementation, 1,130 therapists were eligible to start using the preference elicitation tool. By the end of the post-implementation period, 728 of these therapists (64.4%, *intention to try*) had completed the e-learning on the preference elicitation tool. Of these, 466 therapists (64.0%) completed the e-learning during the initial nine-month implementation period.

Among the 728 therapists who completed the e-learning, 325 therapists (44.6%, *action to try*) used the preference elicitation tool at least once before the end of the post-implementation period. Additionally, 214 of 325 therapists (65.8%) used the tool more than once during the study period. Therapists who started using the tool used it a total of 1,333 times (median use per therapist = 3, IQR = 1–5) with 1,091 unique patients (median use per patient = 1, IQR = 1–1). The median timing of the first use of the tool within a patient’s treatment trajectory was twelve days after treatment start (IQR = 4–35). Table 2 provides an overview of therapist characteristics. A detailed overview of tool usage throughout the study period is shown in Supplementary Figure 6.

**Table 2.** Demographic characteristics of therapists.

	Therapists eligible to use the PE-tool	Therapists who completed e-learning on the PE-tool	Therapists who used the PE-tool at least once
	*n* = 1,130	*n* = 728	*n* = 325
Age in years			
Mean (SD)	44.0 (12.2)	44.7 (11.9)	46.3 (11.3)
Sex			
Female, n (%)	655 (58.0)	439 (60.3)	204 (62.8)
Years affiliated with Chronic CareNet			
Mean (SD)	5.2 (3.3)	5.3 (3.3)	5.3 (3.5)

Abbreviations: n: number; PE-tool: preference elicitation tool; SD: standard deviation.

#### Reach

The 325 therapists who started using the preference elicitation tool used it with 428 patients out of 1,130 patients who started a new treatment trajectory during the first six months post-implementation. This resulted in a reach of 37.9%, indicating that therapists who initiated use of the preference elicitation tool used it with approximately one-third of eligible patients. Table 3 shows baseline characteristics of eligible patients and patients with whom the preference elicitation tool was used.

**Table 3.** Demographic baseline characteristics of patients.

	Patients eligible to use the PE-tool with^a^	Patients with whom the PE-tool was used^a^
	*n* = 1,130	*n* = 428
Age in years		
Mean (SD)	70.2 (10.3)	69.5 (9.4)
Sex		
Female, n (%)	433 (38.3)	162 (37.9)
BMI (kg/m^2^)	*n = 718*	*n = 418*
Mean (SD)	26.9 (4.5)	27.2 (4.5)
Smoking status	*n = 811*	*n = 419*
Current, n (%)	311 (38.4)	150 (35.8)
Former, n (%)	422 (52.0)	206 (49.2)
Never, n (%)	78 (9.6)	63 (15.0)
FWD in meters^b^	*n = 795*	*n = 419*
Median (IQR)	230 (133–390)	213 (130–340)
MWD in meters^b^	*n = 795*	*n = 419*
Median (IQR)	346 (206–537)	330 (217–520)
HRQoL (6–24)^c^	*n = 926*	*n = 419*
Mean (SD)	15.4 (4.1)	15.1 (3.9)

Abbreviations: FWD: functional walking distance; BMI: body mass index; HRQoL: health related quality of life; IQR: interquartile range; MWD: maximal walking distance; n: number; PE-tool: preference elicitation tool; SD: standard deviation.

^a^ Data for the two patient groups were extracted from different databases.

^b^ FWD and MWD were assessed using a standardized treadmill test.

^c^ HRQoL was measured using the Vascular Quality of Life Questionnaire-6 (VascuQoL-6).

### Qualitative assessment (barriers and facilitators)

In total, 30 therapists were invited and eleven agreed to participate. Three therapists indicated they did not have time, and no response was received from others. Characteristics of the eleven therapists who participated in the interviews are shown in Table 4. All therapists worked in primary care practices. Two therapists completed a master’s degree in geriatric physical therapy. Three therapists did not start using the preference elicitation tool after completing the e-learning (i.e. no adoption). Four therapists demonstrated low to medium adoption and used the tool one to four times before the interview. The remaining four therapists showed high adoption and used the tool more than ten times, with one therapist using it over twenty times.

**Table 4.** Demographic characteristics of participating therapists.

Nr.	Sex	Age (years)	Work experience as PT (years)	Master’s degree	Nr. of colleagues with CCN affiliation	Duration of CCN affiliation (years)	Nr. of patients with IC treated annually	Frequency of using PE-tool^a^
T1	Female	40–43	16–20	Yes	1	≥5	9–12	12
T2	Female	36–39	16–20	No	2	≥5	5–8	2
T3	Female	40–43	11–15	No	1	<5	5–8	3
T4	Male	40–43	11–15	No	1	<5	9–12	0
T5	Female	40–43	6–10	No	0	<5	9–12	21
T6	Male	44–47	1–5	No	0	<5	5–8	1
T7	Male	56–59	21–25	No	0	≥5	9–12	14
T8	Male	28–31	6–10	No	3	≥5	9–12	0
T9	Female	24–27	1–5	No	3	<5	17–20	0
T10	Male	24–27	1–5	No	3	<5	1–4	4
T11	Female	36–39	11–15	Yes	1	<5	13–16	11

Abbreviations: CCN: Chronic CareNet; IC: intermittent claudication; Nr: number; PE-tool: preference elicitation tool.

^a^ Frequency of preference elicitation tool use prior to the interview.

We identified 39 barriers and 37 facilitators from the interviews that influenced therapists’ adoption of the preference elicitation tool across all seven domains of the TICD framework. Barriers and facilitators were identified within 25 of 57 potential determinants in the framework. In Table 5, an overview of identified barriers and facilitators is presented. A more detailed explanation of barriers and facilitators that warrant further clarification or present contrasting perspectives is provided below. Supplementary File 2 presents quotations representing the qualitative data.

**Table 5.** Overview of identified barriers and facilitators.

Domain 1: Perceived factors related to the preference elicitation tool		
Determinant	Barriers	Facilitators
**Feasibility**	Excessive amount of features Childish visual design Nonintuitive slider functionality Technical limitation in the navigation Too time-consuming	Intuitive interface Aesthetic visual design Practical slider functionality Easy to use Acceptable time investment
**Accessibility**		Accessible to a broad range of patients
**Compatibility**	Conflicts with already demanding start of treatment Integrating one-on-one time for periodic evaluations disrupts patient training schedules	Naturally complements start of treatment processes Naturally complements periodic evaluation processes

**Observability^a^**	Lack of added value	Structured treatment process Increased patient engagement Increased patient motivation More in-depth conversations
	
	
	
Domain 2: Perceived factors related to the individual therapist		
Determinant	Barriers	Facilitators
**Domain knowledge**	Lack of knowledge in addressing medication adherence or nutrition Lack of motivational interviewing skills	Knowledge of intermittent claudication treatment Motivational interviewing skills

**Awareness and familiarity with the innovation**	Lack of awareness of the tool	
**Knowledge about own practice**	Belief current practice of shared decision-making is sufficient	
**Skills needed to adhere**		No advanced skills needed
**Agreement with the innovation**	Lack of agreement with elements of the tool	Agreement with the tool’s overarching purpose
**Expected outcome^a^**	Fear of losing personal connection Concerns about negative impact on therapeutic relationship Lack of belief in added value for all patients	Expected support in collaborative goal-setting
**Intention and motivation**	Negative first experience with using the tool Lack of intrinsic motivation	Positive experience with the POFs Motivation to provide high-quality patient care Participation in scientific research
**Self-efficacy**	Lack of confidence	Belief in ability
**Learning style**		Comfort in learning by doing E-learning for tool introduction
**Nature of the behavior**	Discomfort with formal communication setting Feeling forced to discuss lifestyle aspects prematurely Conflicts with activity-oriented role perception Lack of degree of automaticity	Familiarity with addressing the tool’s aspects Openness with prioritizing time for patient-centered communication Comfort with joint tool usage
**Capacity to implement change**	Difficulty of meeting competing demands	Ability to use the tool in a flexible way Ability to adapt existing routines Managing patient expectations
Domain 3: Perceived factors related to the patient		
Determinant	Barriers	Facilitators
**Patient needs**	Lack of patient need for additional support	Patient need for additional support
**Patient beliefs and knowledge**	Lower cognitive abilities	Higher cognitive abilities
**Patient preferences**	Lack of patient preference for addressing lifestyle aspects Lack of patient preference to engage in shared decision-making Patient preference for prompt training start	Patient openness to address lifestyle aspects Patient preference to engage in shared decision-making
**Patient behavior**	Patients elaborating extensively	
Domain 4: Perceived factors related to professional interactions		
Determinant	Barriers	Facilitators
**Communication and influence**	Communication overload by CCN Lack of positive peer endorsement	Shared success stories Positive peer endorsement
Domain 5: Perceived factors related to incentives and resources		
Determinant	Barriers	Facilitators
**Availability of necessary resources**	Lack of time in consultation Lack of technological devices in training area	Extra consultation time provided by employer
**Information system**	Lack of integration between CCN system and EHR Inefficient consent process	
Domain 6: Perceived factors related to the organization		
Determinant	Barriers	Facilitators
**Regulations, rules, policies**	Data collection requirements from CCN	Clear directive from CCN
**Priority of desired change**	High workload within general physical therapy	
Domain 7: Social, political and legal factors		
Determinant	Barriers	Facilitators
**Financing policies**	Limited reimbursement rates by health insurance	

Abbreviations: CCN: Chronic CareNet; HER: electronic health record; PE-tool: preference elicitation tool; POFs: personalized outcomes forecasts.

^a^Observability refers to the effects therapists have actually experienced when using the tool, whereas expected outcome relates to therapists’ anticipated effects before tool use.

### Perceived factors related to the preference elicitation tool

Therapists emphasized the tool’s intuitive interface, which allowed them to easily navigate through its features. However, some therapists found the number of features excessive and making it feel overwhelming. Several therapists appreciated the tool’s clarity and noted it made the tool accessible to a broad range of patients, whereas other therapists perceived the design as overly simplistic and somewhat childish. Similarly, opinions on the slider functionality for eliciting patient preferences varied. Some therapists described it as nonintuitive, whereas others found it highly practical, provided they actively guided patients in understanding and using the slider.

All therapists highlighted the time investment required to use the tool and generally believed it did not save time. Some found the tool excessively time-consuming, which was seen as conflicting with the already demanding start of treatment due to tasks such as baseline outcome measurements. Others considered the time investment acceptable, as it was manageable within standard consultation time. Certain therapists perceived the tool as incompatible with periodic evaluations, since incorporating extensive one-on-one sessions disrupted patients’ training schedules. Conversely, others mentioned the tool complemented the natural course of treatment, as conversations about the treatment plan typically arose during initial consultations and periodic evaluations.

Therapists who discontinued using the tool after an initial trial felt it lacked observable value compared to their current practice, as it did not offer new information or improve the patient conversation. In contrast, therapists who continued using the tool experienced observable benefits such as a more structured treatment process, more in-depth conversations and increased patient engagement. For these therapists, the tool made it easier to collaborate genuinely with patients and prevented the making of decisions on their behalf.

### Perceived factors related to the individual therapist

The agreement of therapists with the tool’s overarching purpose of enhancing shared decision-making and patient engagement facilitated their acceptance of the tool. Nevertheless, some therapists perceived their current practice already incorporated these elements sufficiently, and thus saw little incentive to change. Difficulty of meeting all competing work-related demands also limited therapists’ motivation and capacity to adopt innovations. Conversely, other therapists were encouraged to use the tool by external motivators. For example, participation in this study reinforced motivation to actively engage with the tool, or therapists were curious based on prior positive experiences with the POFs.

Most therapists who demonstrated little or no adoption of the tool expressed discomfort with the behavior required to use it. Therapists disliked the necessary formal communication setting of the tool, which contrasted with their preference for discussing treatment decisions more informally, such as during treadmill exercises. The added dialogue required to use the tool also led to a delay in the start of exercise therapy. This conflicted with therapists’ activity-oriented role perception, as they viewed their primary role as engaging patients in physical activity. Moreover, some therapists expressed concern, although not necessarily based on actual experience, that using the tool might weaken the personal connection with patients due to the increased computer use. Furthermore, therapists preferred to discuss lifestyle aspects when they deemed appropriate, but felt the prominent display of these aspects in the tool forced them to address lifestyle prematurely. Some therapists thought that imposing lifestyle discussions might frustrate patients, potentially damaging the therapeutic relationship. Overall, therapists perceived that use of the tool lacked automaticity; it had not yet become part of their regular routines, which meant it was sometimes forgotten or not actively considered during consultations.

In contrast, therapists who continued using the tool found its focus on aspects like physical activity and lifestyle familiar, as these were considered the key pillars of intermittent claudication treatment. Therapists reported being open to invest extra time to build trust with patients. The joint tool usage aligned with their preference for patients taking more of the lead rather than them following their instructions, and made therapists feel comfortable with the communication setting. Therapists described using the tool flexibly, tailoring its use to individual patient needs or treatment contexts. For example, they selected specific parts or spread its use across multiple consultations. Others adapted routines, for instance by scheduling a consultation specifically to address the tool, or prioritized time within consultations. Managing patient expectations by clear communication about the tool’s purpose was found to facilitate this process, as it increased patient understanding and openness towards the tool.

### Perceived factors related to the patient

Therapists perceived the tool as unnecessary for patients who were already motivated or had clear goals, as they did not need additional support. Conversely, therapists believed that patients with limited self-management skills could benefit from the additional support, making them more likely to use the tool. In particular, therapists highlighted the value of the tool’s printed summary of the treatment plan as helpful guide for these patients. Therapists were less enthusiastic about using the tool for patients with lower cognitive ability or those elaborate excessively on questions, since it required additional effort or time to complete the tool. Therapists also were reluctant to use the tool with patients whom they perceived as preferring to start training promptly, or who had limited interest in engaging in shared decision-making or discussions about their lifestyle choices. However, when therapists felt that patients were open to shared decision-making or discussing lifestyle issues, they were more inclined to use the tool, as it then aligned with the patient’s stage of behavior change.

### Perceived factors from other domains

Limited consultation time was cited as hindering tool adoption, as some therapists felt it constrained their ability to use the tool effectively alongside other tasks. One therapist noted his employer allowed to extend the initial consultation, which enabled tool integration. Some therapists mentioned the lack of integration between the Chronic CareNet portal and electronic health records added unnecessary administrative burden. Therapists also mentioned Chronic CareNet’s clear directive regarding the tool. While some perceived the directive to start using the tool came across as mandatory, they found it ultimately supported their engagement. One therapist assumed the tool as standard part of practice after joining Chronic CareNet and began using it accordingly. Lastly, one therapist, a practice co-owner, highlighted the low reimbursement rates as broader system-level challenge. He felt the limited reimbursement rates made running a practice more challenging, which indirectly hindered his capacity to change.

## Discussion

This study examined the adoption and reach of a preference elicitation tool for patients with intermittent claudication, as well as physical therapists’ barriers and facilitators for adopting the tool. Our findings show that 64% of eligible therapists completed the e-learning, and 45% of those who completed the e-learning began using the tool in their practice. Therapists used the tool with 38% of eligible patients. Therapists’ adoption of the tool was influenced by various factors. Identified barriers included the required time investment, discomfort with the formal communication setting, and conflicts with activity-oriented role perceptions. Facilitators included the tool’s intuitiveness and user-friendliness, observable benefits such as increased patient engagement, and flexible use of the tool by therapists.

One year after the nine-month implementation period, over half of eligible therapists completed the e-learning on the preference elicitation tool. About half of these therapists – roughly a quarter of total eligible therapists – proceeded using the tool in practice, which highlights a substantial drop-off between interest and actual use. Compared to the POFs implementation, during which 51% of therapists began using the tool in practice, the adoption of the preference elicitation tool appears to be relatively modest [22]. Following Rogers’ Diffusion of Innovations theory, the e-learning completion rate indicates quite successful progression through the knowledge stage, while the lower utilization rate suggests selective transition of therapists through the persuasion and decision stages [30]. This discontinuation may have stemmed from uncertainty about the tool’s compatibility or relative advantage in practice [30], as also mentioned by interviewed therapists. The position of the preference elicitation tool as the second innovation introduced within a relatively short timeframe, following the introduction of POFs in late 2019, may also have contributed to the modest adoption. The repeated introduction of novel tools could have led to ‘change fatigue’, due to therapists feeling the constant need to adapt and advance clinical practice [31]. This effect may have been exacerbated by the current climate in the healthcare sector, where high workload and system strain might leave limited capacity to embrace new initiatives [32].

Therapists used the preference elicitation tool with about one-third of eligible patients, indicating its use is not consistently embedded across all patients with intermittent claudication. Interviewed therapists reported several patient-related factors potentially explaining this variability. The tool was seen as particularly beneficial for patients who are uncertain about their treatment goals or have limited self-management skills. Conversely, the tool was considered less useful for motivated patients or those with strong preconceived treatment ideas. Other patient-related barriers included perceived patient disinterest in shared decision-making and lower cognitive abilities, common obstacles to decision aid uptake [16,33–35]. Therapists primarily used the tool with patients they deemed most likely to benefit, which may have limited the tool’s reach. This tendency to look for a specific patient fit aligns with broader findings of healthcare professionals screening patients a priori when using shared decision-making or decision aids, potentially excluding patients who might still benefit [36]. Therefore, future research should explore patients’ actual perceptions of the preference elicitation tool.

Our qualitative findings highlight the multifaceted factors influencing therapists’ adoption of the preference elicitation tool and underscore the complexity of implementing decision aids [37]. Barriers mentioned by non-adopters, such as the tool being time-consuming or offering limited added value compared to their current shared decision-making practices, align with earlier studies that identified time constraints and a limited perceived need for decision aids as common obstacles to adoption [16,36,38–40]. Interestingly, evidence suggests a discrepancy between perceived and actual levels of shared decision-making [41–43], raising the possibility that the tool’s potential benefits are not fully recognized. Although concerns about the time-investment were common, our previous trial analyzing 72 consultations involving tool usage showed an average consultation duration of 29.8 min (SD 11.4), which falls within standard consultation time [15]. The sense of time pressure appeared to be shaped more by therapists’ role expectations and competing priorities, such as starting exercise therapy quickly. This perception reflects prior findings of physical therapists tending to view themselves as ‘doers’ rather than ‘talkers’, potentially hindering shared decision-making and person-centered care [44–47]. Conversely, adopting therapists were motivated to start using the tool and recognized the tool’s potential to improve the quality of care. Their flexible use of the tool enabled integration into routines and reduced the perceived burden. A positive user attitude and improvements in the clinical process are frequently cited as key facilitators for the uptake of decision aids and shared decision-making [36,48,49]. Perhaps, the adaptability and flexibility demonstrated by adopting therapists enabled them to experience the tool’s benefits more fully [50,51]. Nonetheless, recognizing flexible tool use as an option of remains essential, as this may not have been fully considered by non-adopters.

Although similar tools to the preference elicitation tool are currently lacking in physical therapy, studies in other settings show comparable implementation challenges. For example, Rake et al. [35] found that medical specialists valued how an encounter decision aid using option grids improved the dialogue but struggled with its fit into routine care, information overload, and use with patients deemed unsuitable. As also indicated by adopting therapists in our study, Rake et al. reported customized use and workflow flexibility as facilitators. Claessens et al. [52] found that primary care practice nurses perceived a tool for visualizing patients’ burden of disease as too time-consuming relative to its perceived benefits, particularly because it required changes to current routines and some patients were perceived unmotivated to take an active role in their care. Yet both studies emphasize the potential of such tools to improve clinician-patient conversations. These findings align with ours: while the preference elicitation tool is considered valuable for enhancing person-centered communication, successful implementation depends on a combination of factors, including therapists’ ability to embed the tool in practice, its compatibility with existing routines, and its perceived applicability to a diverse range of patients.

## Implications for practice and future research

Our findings underscore that successful adoption and sustainable implementation extend beyond the dissemination of the preference elicitation tool and its accompanying e-learning [16,53,54]. Future implementation efforts should focus on increasing therapists’ awareness of the tool’s flexibility and providing practical strategies for integrating it into different workflows. Learning from peers and sharing best practices may facilitate this process, alongside ensuring the tool’s layout intuitively reflects its flexibility [30,43]. Furthermore, maintaining an open dialogue about therapists’ beliefs regarding shared decision-making and using the tool is important. As decision aids support, not replace, shared decision-making, some therapists may already feel they effectively engage in shared decision-making without the tool. Open discussions and reflective practice assessments could affirm the validity of current approaches or uncover misconceptions, fostering constructive conversations about how shared decision-making can be further enhanced [55].

Future research should explore factors that may influence the implementation of the preference elicitation tool beyond the individual therapist, such as the evolving role of the physical therapy profession. Specifically, it should be explored how the shift from activity-focused care to person-centered care is currently understood, the extent to which it is implemented in daily physical therapy practice, and how this shift is supported or hindered. Patient- and context related factors should also be explored, including preferences regarding shared decision-making and the tool itself, as well as the tool’s accessibility for patients with limited health literacy. Additionally, it is important to assess whether use of the tool influences the content of and adherence to SET programs. For example, through patient interviews or by comparing the adherence to treatment trajectories between patients with whom the tool was used and those with whom it was not. These insights will inform the development of tailored implementation strategies to further enhance adoption and sustainable use in clinical practice.

## Strengths and limitations

Strengths of our study include the multi-method approach and the use of the TICD framework, which enabled us to obtain comprehensive insights into the implementation of the preference elicitation tool and consider a wide range of implementation factors. Limitations include limited generalizability, as we did not include therapists who did not complete the e-learning on the preference elicitation tool, which may have resulted in missed barriers for the uptake of the tool. Furthermore, our study focuses solely on therapists’ perspectives, without considering patient viewpoints. As a result, identified patient-related factors are based only on therapists’ perceptions, leaving it unclear how patients actually perceive the tool and its impact on their care. Also, although data saturation was achieved, the sample size of eleven therapists was quite small. Nevertheless, research confirms that for relatively homogeneous study populations, saturation can typically be reached with nine to seventeen interviews [56]. Lastly, the involvement of research team members in the tool development may have introduced bias in the interpretation of the qualitative results. To mitigate this potential, bracketing sessions were conducted and all coded interview transcripts were reviewed by one researcher who had no prior involvement in the tool’s development.

## Conclusions

This study is the first to evaluate an encounter decision aid within the physical therapy treatment of patients with intermittent claudication. Moderate adoption and reach rates indicate opportunities for improvements. This study highlights the complexity of therapists’ decision-making in (non)adopting the tool in clinical practice, as exemplified by a wide range of often contrasting barriers and facilitators. Addressing key barriers, such as providing therapists with practical strategies to integrate the tool into their workflow and fostering open dialogues and reflective practice around shared decision-making, could enhance implementation. Future research should examine factors influencing implementation beyond the individual therapist, explore patient perspectives on the tool, and develop tailored implementation strategies to enhance adoption and sustainability. Ultimately, successful implementation of shared decision-making and decision aids requires a tailored approach, as both therapists and patients have unique needs and preferences.

## Supplementary Material

Supplemental Material

## Data Availability

Deidentified data used and/or analysed during the current study are available from the corresponding author on reasonable request.
