# Supplementary material for: A guideline-based preference elicitation tool to enhance shared decision-making in supervised exercise therapy for patients with intermittent claudication: a process evaluation
Source: Ann Med. 2025 Aug 4;57(1):2540022. doi: 10.1080/07853890.2025.2540022 (PMC12322985; doi:10.1080/07853890.2025.2540022)
Supplement: Supplemental Material [file IANN_A_2540022_SM5047.zip › suppl_data/20250328 Supplementary files 1 and 2.docx]

**SUPPLEMENTARY FILE 1. Interview guide.**

Notes for the interviewer:

- Begin by introducing yourself (name and job function) and providing context for the research in which this interview takes place;

- For therapists who **have used the preference elicitation tool in practice**, all questions are applicable. For therapists who **have only completed the e-learning**, questions about experiences with tool use should focus on expectations, perceptions, and reasons for (not) using the tool.

**1. Introduction and background**

- Could you briefly describe who you are and your current professional situation?

- What makes a treatment trajectory for patients with intermittent claudication successful for you?

- What is your perspective on shared decision-making in physical therapy?

**2. Perspectives and experiences with the preference elicitation tool**

- To what extent do you use the preference elicitation tool?

- What made you (not) start using the preference elicitation tool? What made you (not) continue using it?

- How do you experience using the preference elicitation tool?

- (How) does the preference elicitation tool fit into your current practice of intermittent claudication treatment?

- (How) does using the preference elicitation tool contribute to your work as a physical therapist or intermittent claudication treatment?

**3. Integration into practice**

- What effort was required to learn how to work with the preference elicitation tool?

- What effort was required to implement the preference elicitation tool into your clinical practice?

- To what extent do you feel competent when using the preference elicitation tool?

**4. Patient factors**

- To what extent do you consider the preference elicitation tool eligible for use with all patients with intermittent claudication?

- How well does the preference elicitation align with patient needs or preference, in your perception?

**5. Tool use and influencing factors**

- To what extent does your work environment influence your use of the preference elicitation tool?

- To what extent do you (not) intend to continue/start using the preference elicitation tool?

- What other factors encourage or discourage you to continue/start using the preference elicitation tool?

**6. Wrap-up**

- What do you consider most important barriers or facilitators for (not) using the preference elicitation tool?

- Do you have any additional points to mention that were not covered during the interview?

**SUPPLEMENTARY FILE 2. Overview of identified barriers and facilitators including illustrative quotations.**

| **Domain 1: Perceived factors related to the preference elicitation tool** | | | | |
| --- | --- | --- | --- | --- |
| **Determinant** | **Barrier** | **Quote** | **Facilitator** | **Quote** |
| **Feasibility** | **Excessive amount of features** | *You have to click quite a lot, search and scroll. […] It keeps getting longer, right. First you had the POFs, then the PE-tool. It’s, how do you say… it becomes less clear because of that. (T7)* | **Intuitive interface** | *It’s very straightforward. Just follow the steps, give an answer, and click the arrow. (T11)* |
|  | **Childish visual design** | *I found it too school-like, a little too childish. (T3)* | **Aesthetic visual design** | *It actually looks really nice. (T4)* |
|  | **Nonintuitive slider functionality** | *The slider functionality [to elicit patient preferences] seems very easy, but patients often don’t understand it very well. They find that difficult. (T5)* | **Practical slider functionality** | *That slider mechanism [to elicit patient preferences] is the most practical and easiest part of the tool. It makes it easier for patients to set priorities. (T10)* |
|  | **Technical limitation in the navigation** | *It lagged, the scrolling in the first part was unmanageable, it jumped straight to the bottom of the screen. (T6)* | **Easy to use** | *It’s really user-friendly. You move through it easily. (T2)* |
|  | **Too time-consuming** | *It takes a lot of time to fill everything out, so you might not complete all the steps. (T5)* | **Acceptable time investment** | *For me, the time commitment was not a problem. I believe it only took one treatment session to get through it. (T6)* |
| **Accessibility** |  |  | **Accessible to a broad range of patients** | *It’s clear, especially for people with low health literacy. Visually, it’s easy to understand, making it usable to a wide range of patients, including older people. (T9)* |
| **Compatibility** | **Conflicts with already demanding start of treatment** | *I think it might just be that in the initial phase of treatment, so much needs to be done all at once. It really feels like a battery of tasks in the beginning. […] The tool is ultimately just a means, but this means ends up becoming bigger than the goal. (T7)* | **Naturally complements start of treatment processes** | *I primarily use it at the start of treatment. Then you’re still in that initial phase. Once I’ve gathered all the measurements and related information, I have a conversation with the patient using the tool to see if they… if they have any ideas about what is important to them. (T1)* |
|  | **Integrating one-on-one time for periodic evaluations disrupts patients’ training schedules** | *By the time evaluations come around, patients are usually more independent in their training. I often have several patients training together at the same time or overlapping sessions, which makes it difficult to leave the training area for a dedicated conversation in the treatment room. (T1)* | **Naturally complements periodic evaluation processes** | *When patients return for an evaluation, you go over the measurements again, which naturally leads to reviewing and discussing the treatment plan. That’s when you come across the tool again. (T6)* |
| **Observability** | **Lack of added value** | *It didn’t provide me with any new information. I’ve had these kinds of conversations with the patient before. There was no new information for me or for the patient either. (T3)* | **Structured treatment process** | *It’s also a kind of ‘reminder’ for me as therapist to really think things through. When you see another patient every half hour, things move quickly, and I tend to make treatment decisions on the spot, and don’t really make a, you know, plan-plan. You sometimes skip steps or overlook things that are important for the patient. When you create a plan in such a structured way, those issues are less likely to happen. So, the structure is really helpful. (T5)* |
|  |  |  | **Increased patient engagement** | *Before, I would just make those decisions on my own. Now, by involving the patient in the process, having them think along with you, and showing it on the screen, I feel they become more engaged. They actively participate by helping to fill out the questions, which makes it feel more real and helps them retain the information better. (T11)* |
|  |  |  | **Increased patient motivation** | *It’s also about patient motivation. With patients I’ve used the tool with, they immediately know, ‘This is what we’re going to do, and this is how we’re going to do it.’ You can really see they are more committed. It’s as if they’re behaving in a healthier or more active way, which is different from patients who weren’t involved in creating the treatment plan like this. (T10)* |
|  |  |  | **More in-depth conversations** | *You now dive deeper into aspects like lifestyle, as well as the training both under my supervision and done independently by the patient. I mention these aspects more thoroughly now. Normally, I probably would have mentioned them less; we would simply just do it. (T1)* |
| **Domain 2: Perceived factors related to the individual therapist** | | | | |
| **Determinant** | **Barrier** | **Quote** | **Facilitator** | **Quote** |
| **Domain knowledge** | **Lack of knowledge in addressing specific topics (e.g., medication adherence, nutrition)** | *If I have a patient where there’s a lot to be gained in terms of nutrition… how do I approach that conversation? I haven’t encountered many cases like that. So, I need to explore that part further. (T6)* | **Knowledge of intermittent claudication treatment** | *You need to have a solid understanding of the condition, of the evidence-based treatments that are available. […] So you can give examples that you naturally incorporate when using the tool. (T6)* |
|  | **Lack of motivational interviewing skills** | *Motivational interviewing will probably help me more with these patients, and I think I might be missing that a bit. (T9)* | **Motivational interviewing skills** | *I do think motivational interviewing really adds value for these kind of decisions, because someone has to make lifestyle changes. You need that to complete the tool properly. Otherwise, you just move the sliders and that’s it, without actually doing anything with it. (T3)* |
| **Awareness and familiarity with the innovation** | **Lack of awareness of the tool** | *If we hadn’t had this interview, I don’t think I would have thought about it anymore. I lost sight of it. (T8)* |  |  |
| **Knowledge about own practice** | **Belief current practice of shared decision-making is sufficient** | *I already bring it up during the intake, you know, shared decision-making and explaining what people can expect. So yes, I’m actually already doing it in a fixed way, which made me think, well, I like it this way, so I’ll keep doing it like this because it works. (T9)* |  |  |
| **Skills needed to adhere** |  |  | **No advanced skills needed** | *I don’t think you need extra skills. At least, that’s just the basic level that I hope every physical therapist has. (T11)* |
| **Agreement with the innovation** | **Lack of agreement with elements of the tool** | *There are a few things where I sometimes think, hmm, does it all need to be so detailed? For example, how intensive the training should be –you can give five options. Honestly, people have no idea. (T1)* | **Agreement with the tool’s overarching purpose** | *Patients are used to the idea that as a healthcare provider, you’re there to help them get rid of their complaints. But with conditions like this, which they have to learn to cope with for the rest of their lives, you actually want them to reach a point where they take control themselves. So you also want the patient to be actively involved in their treatment. (T10)* |
| **Expected outcome** | **Fear of losing personal connection** | *Maintaining a connection with the patient is really important to me. It’s easy to get caught up in the paperwork, but I find it more important to maintain that relationship. I’m afraid that if I focus too much on the tool, I’ll lose the trust and rapport we’ve built, just to complete it. For me, maintaining trust and the relationship is more important than finishing the tool. […] I’m afraid of losing that personal connection, and that ultimately, it negatively affects treatment outcomes after two, three, four, or five months. (T7)* | **Expected support in collaborative goal-setting** | *Patients often struggle to break down their main goal into smaller steps and then take action. Like, ‘If I do this activity this often, I’ll reach my goal.’ […] I think it can really help in setting goals together and discussing how to achieve those goals. (T4)* |
|  | **Concerns about negative impact on therapeutic relationship** | *I feel like it imposes behavioral change, which goes completely against my principles. Behavioral change involves a lot of emotion and discussing choices people have made, knowing they weren’t the healthiest. I don’t want to judge them for that. When I immediately present them with a screen or a piece of paper showing those lifestyle factors and ask, ‘What do you want to change?’, I’m really afraid they’ll dig in their heels, and it will create distance between us. (T3)* |  |  |
|  | **Lack of belief in added value for all patients** | *You can always use the tool, but I doubt it’s added value is there for everyone, I’m hesitant to say it is. (T6)* |  |  |
| **Intention and motivation** | **Negative first experience with using the tool** | *I think the first time using the tool was decisive for not using it anymore afterwards. It was just disappointing. (T3)* | **Positive experience with the POFs** | *I wanted to see what it was about. Those graphs, the POFs, I find them very valuable. So, yeah, that’s why I was also curious to see what else was included. (T2)* |
|  | **Lack of intrinsic motivation** | *There are, of course, many other things to do, and yeah, it’s that intrinsic motivation that’s just not strong enough. (T4)* | **Motivation to provide high-quality patient care** | *As a therapist, I always want to provide the best possible care for patients, so I thought it was worth trying. Because I want to provide high-quality care, I wanted to give it a chance. (T3)* |
|  |  |  | **Participation in scientific research** | *Participating in that observational study served as a prompt for me to actually start using it. […] I think if I hadn’t participated in those observations, I would have applied it less thoroughly. (T1)* |
| **Self-efficacy** | **Lack of confidence** | *I was still quite clumsy with it [the tool], because I didn’t exactly know in advance which icon to use for what questions or how to use it correctly. So, I was inexperienced, and yes, that’s not the tool’s fault but also due to my own use of it. (T6)* | **Belief in ability** | *The more you use it, the easier it gets. Since I’ve been using the tool from the start, it already feels quite easy for me. (T1)* |
| **Learning style** |  |  | **Comfort in learning by doing** | *Just go ahead and do it. You have to follow those steps and see how it works. Of course, the first time, I did think, ‘How should I ask this question to the patient?’ But that’s something you have to figure out with all other questionnaires as well. I either explain it, or the patient reads it and responds. I just started using it. (T11)* |
|  |  |  | **E-learning for tool introduction** | *I think it's really good that such a course is available, and that it's easy to do. It's an easy way to make it visible, to get acquainted with it. (T3)* |
| **Nature of the behavior** | **Discomfort with formal communication setting** | *I prefer having these kinds of conversations when patients are on the treadmill, or in between exercises, as it allows for a more relaxed discussion. Right now, we really have to sit down and look at the screen together. I didn’t like it. (T3)* | **Familiarity with addressing the tool’s aspects** | *The tool covers the topics [physical exercise, quit smoking, eating pattern, medication adherence] that you have to discuss anyway, They are the key pillars for this population. So yes, basically, it's always there. (T11)* |
|  | **Feeling forced to discuss lifestyle aspects prematurely** | *These are sensitive topics: weight, smoking, alcohol use. I usually don’t bring up these kinds of conversations within the first three sessions. I get the basic information, like, 'Okay, nutrition is good, smoking still an issue, all right, we know that.' Then I try to first build rapport, establish a relationship before diving into those topics. […] Otherwise you can immediately see them thinking, 'Oh, here we go again.' They don’t say it, but it shows on their faces. So, I’ve decided for myself, 'You know what, we’ll get to that once we’ve seen each other a bit more often. (T3)* | **Openness with prioritizing time for patient-centered communication** | *I don't see it’s a problem that you're talking for a longer time, especially with patients who need a lot of explanation to really motivate them. Then you start in the room, gaining a bit of trust and getting to know them. After that, you start training. So, I don't mind spending extra time behind the computer with someone. (T6)* |
|  | **Conflicts with activity-oriented role perception** | *Look, I enjoy getting patients physically active the most, and then you’ve already spent an hour with the intake, mainly just talking […] So, patients also really want to get started training because that's what they come here for. But then I think, well, that’s my interpretation, you know, that sitting behind the computer again for a second session might be a bit much. (T4)* | **Comfort with joint tool usage** | *You need to be open to letting the patient take more of the lead, rather than directing them and giving the impression that they should simply follow your instructions. (T10)* |
|  | **Lack of degree of automaticity** | *It’s not yet part of my routines to use it, just like the POFs, as a standard with every patient. (T10)* |  |  |
| **Capacity to implement change** | **Difficulty of meeting competing demands** | *Sometimes it’s just easier to skip it for the moment. […] It’s like spinning small plates; if you have five plates you need to keep spinning, you won’t quickly add a sixth one. But if you only have two plates to manage, adding a third or even a fourth is much easier. It’s more about the number of plates you need to keep spinning, I think. (T7)* | **Ability to use the tool in a flexible way** | *I go with the flow of what’s happening and try to integrate that into my practice. That’s basically what I do. If I think, now I can fill in this part of the tool, I’ll do it. But if I think, no, the timing isn’t right, then I’ll leave it and come back to it another time. (T11)* |
|  |  |  | **Ability to adapt existing routines** | *I usually schedule a double intake session lasting an hour. During that intake, I conduct the regular tasks of the intake with the questionnaires and the walking test, and then it depends on how much time is left. Sometimes, I fill in the section of the tool related to training with the therapist. Then, during the next session, we start the training. In the third session, I use the time to complete the rest of the tool and finalize the treatment plan. (T5)* |
|  |  |  | **Managing patient expectations** | *If you clearly explain during the intake that treatment involves more than just walking, and discuss it together, patients will understand that this is part of the treatment. (T1)* |
| **Domain 3: Perceived factors related to the patient** | | | | |
| **Determinant** | **Barrier** | **Quote** | **Facilitator** | **Quote** |
| **Patient needs** | **Lack of patient need for additional support** | *Some patients are so motivated by themselves that they don't really need it. (T4)* | **Patient need for additional support** | *If I notice, especially, that someone is unsure about what they want, I tend to use the tool more quickly. (T10)* |
| **Patient beliefs and knowledge** | **Lower cognitive abilities** | *There are people who simply don’t understand it, which also has to do with their level of understanding. Sometimes, I’m just happy if I can clearly explain the importance of walking training. When I notice that half of what I say doesn’t get through, using the tool adds too much information for these people. As a result, I think the key information doesn’t stick. In those cases, I think I can communicate more effectively by directly telling them what I want, and asking them to repeat it. (T11)* | **Higher cognitive abilities** | *A person with better cognitive abilities will benefit more from using the tool; they will be more likely to recognize the importance of their own role. (T6)* |
| **Patient preferences** | **Lack of patient preference for addressing lifestyle aspects** | *I feel that people are generally less open when it comes to topics like healthy eating or smoking. I believe you need to get to know someone better before addressing those issues. [...] Most people come here asking questions like, ‘Which exercises should I do?’ They’re not seeking help with making healthy lifestyle choices. (T3)* | **Patient openness to address lifestyle aspects** | *One of my patients was willing to train here and also wanted to do some exercises at home. So, I casually brought up alcohol use and smoking, letting it sit with him. Every now and then, I checked in, like, ‘Have you thought about it a bit more?’ When he seemed ready to make a plan for that, that’s when I went ahead using the tool to create a plan. (T5)* |
|  | **Lack of patient preference to engage in shared decision-making** | *For example, I recently saw a man who said, ‘If you tell me I need to work really hard on that, I’ll work really hard on it.’ (T5)* | **Patient preference to engage in shared decision-making** | *I think patients need to get used to the PE-tool, but they may also find it helpful, as it allows them to share their thoughts and highlight what’s important to them. It’s not being decided for them. (T2)* |
|  | **Patient preference for prompt training start** | *I notice that people really want to do something. They say things like, ‘Oh, I didn’t need to bring these clothes,’ or ‘I was hoping we could do something already, or that I could take something home.’ In that way, I notice they feel like, ‘Okay, I’m here to get something out of this. (T9)* |  |  |
| **Patient behavior** | **Patients elaborating extensively** | *I have patients who find it all very difficult or who elaborate a lot on each question you ask. Then, I end up making fewer decisions together because it becomes unmanageable.* *At that point, I realize I just need to say, ‘We’re going to do this.’ (T5)* |  |  |
| **Domain 4: Perceived factors related to professional interactions** | | | | |
| **Determinant** | **Barrier** | **Quote** | **Facilitator** | **Quote** |
| **Communication and influence** | **Communication overload by Chronic CareNet** | *I get a lot of emails from Chronic CareNet. Sometimes there are so many things mixed in. I feel like I get emails a couple of times a week, so it’s easy to overlook them. (T4)* | **Shared success stories** | *The emails from Chronic CareNet about the tool included feedback like, ‘So many therapists are already using it’, with quotes such as ‘It’s easy to use’ and ‘You’re truly having a shared conversation’. When I read that, I think… Yes, I’m going to give it a try too. (T6)* |
|  | **Lack of positive peer endorsement** | *We [my colleague and I] talked about it recently. She mentioned, ‘I don’t do anything with the tool’. […] That reinforced my feeling that my first experience wasn’t very smooth. Well, if she doesn’t use it, I don’t use it. We are influenced by each other. (T3)* | **Positive peer endorsement** | *A colleague mentioned it during a meeting. He said, ‘They now have a new option to create a treatment plan with your patient. […] I think it’s worth checking out.’ (T10)* |
| **Domain 5: Perceived factors related to incentives and resources** | | | | |
| **Determinant** | **Barrier** | **Quote** | **Facilitator** | **Quote** |
| **Availability of necessary resources** | **Lack of time in consultation** | *In my opinion, it takes more time than I currently have. I always schedule an hour for the intake and already use the entire time, so there’s already some time pressure on the consultations. (T9)* | **Extra consultation time provided by employer** | *I really do need extra time for this. […] I arranged with my employer to add an extra half hour at the start of a new trajectory*. (T10) |
|  | **Lack of technological devices in training area** | *We don’t have a computer in the training area, so I can’t use the tool then. Maybe a tablet or something would help, to use the tool on the spot. (T2)* |  |  |
| **Information system** | **Lack of integration between Chronic CareNet system and EHR** | *Then I think, ‘I already entered that in the EHR, and now I have to enter it again in the Chronic CareNet system’. Well, it’s not that much, but it does feel like I’m doing the same thing twice sometimes. (T9)* |  |  |
|  | **Inefficient consent process** | *I really find the hassle with obtaining patient consent and the signature annoying. Everyone is struggling with the mouse to sign. […] It sometimes takes 5 minutes just to get that signature. (T4)* |  |  |
| **Domain 6: Perceived factors related to the organization** | | | | |
| **Determinant** | **Barrier** | **Quote** | **Facilitator** | **Quote** |
| **Regulations, rules, policies** | **Data collection requirements from Chronic CareNet** | *You have to fill out questionnaires to get all the data complete. Then I notice patients thinking, ‘Wow, lots of questions’. And then I actually cut it off. If you then have to ask even more questions… (T2)* | **Clear directive from Chronic CareNet** | *I thought it was just a standard part of practice, so I kind of started using it in that sense. […] I also thought it was, well, mandatory, I think. (T5)* |
| **Priority of desired change** | **High workload within general physical therapy** | *The workload and scheduling pressure is just very high, which makes that step more difficult. […] Yeah, those new things just tend to fall to the bottom of the list sometimes. Then you’re busy with completely different things. (T4)* |  |  |
| **Domain 7: Social, political and legal factors** | | | | |
| **Determinant** | **Barrier** | **Quote** | **Facilitator** | **Quote** |
| **Financing policies** | **Limited reimbursement rates by health insurance** | *The low rates just make everything, it makes things more challenging. I am co-owner of a practice, and there aren’t many investment opportunities. All of these things add up, which, well, is part of running a business, but it also makes working as a physical therapist, you know, sometimes harder. […] So, indirectly, it really does have an impact. (T4)* |  |  |
