# Supplementary material for: A guideline-based preference elicitation tool to enhance shared decision-making in supervised exercise therapy for patients with intermittent claudication: a process evaluation
Source: Ann Med. 2025 Aug 4;57(1):2540022. doi: 10.1080/07853890.2025.2540022 (PMC12322985; doi:10.1080/07853890.2025.2540022)
Supplement: Supplemental Material [file IANN_A_2540022_SM5047.zip › suppl_data/Legends to the supplementary figures.docx]

**Legends to the supplementary figures**

**Supplementary Figure 1.** First part of the preference elicitation tool in which treatment options are discussed and the patient’s preferences regarding these options can be elicited.

**Supplementary Figure 2.** Printable version of the part of the preference elicitation tool in which the patient’s preferences regarding treatment options can be elicited.

**Supplementary Figure 3.** Part of the preference elicitation tool (in this image partially filled out) in which the patient’s preferences regarding the treatment options are elicited and treatment options can be prioritized.

**Supplementary Figure 4.** Part of the preference elicitation tool in which detailed patient preferences regarding supervised training can be elicited.

**Supplementary Figure 5.** Part of the preference elicitation tool in which therapists can generate a printable summary.

**Supplementary Figure 6.** Use of the preference elicitation tool throughout the study period.
