# Supplementary figures and images for: A guideline-based preference elicitation tool to enhance shared decision-making in supervised exercise therapy for patients with intermittent claudication: a process evaluation
Source: Ann Med. 2025 Aug 4;57(1):2540022. doi: 10.1080/07853890.2025.2540022 (PMC12322985; doi:10.1080/07853890.2025.2540022)

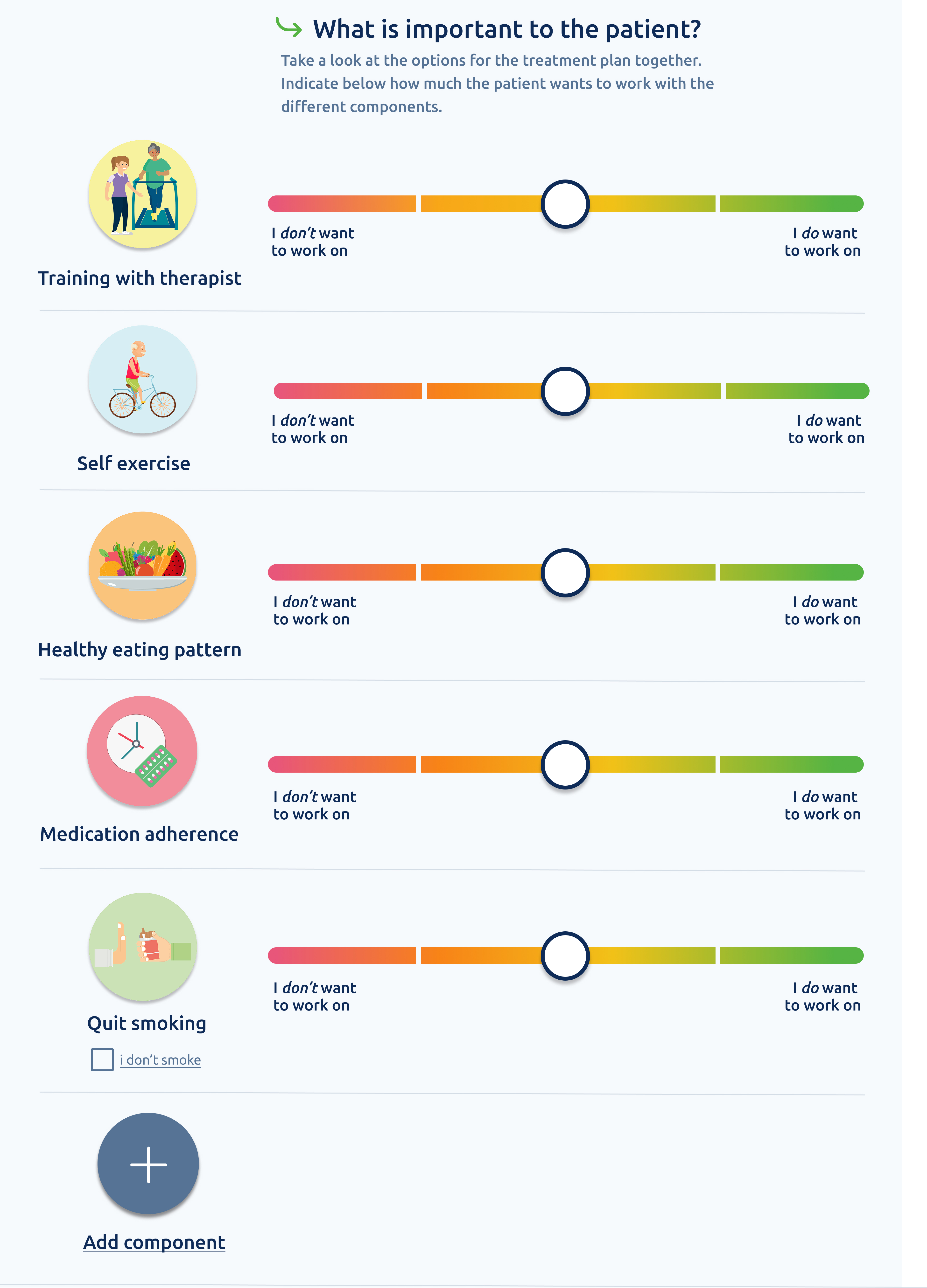

Supplement: Supplemental Material [file IANN_A_2540022_SM5047.zip › suppl_data/Supplementary Figure 1.jpg]

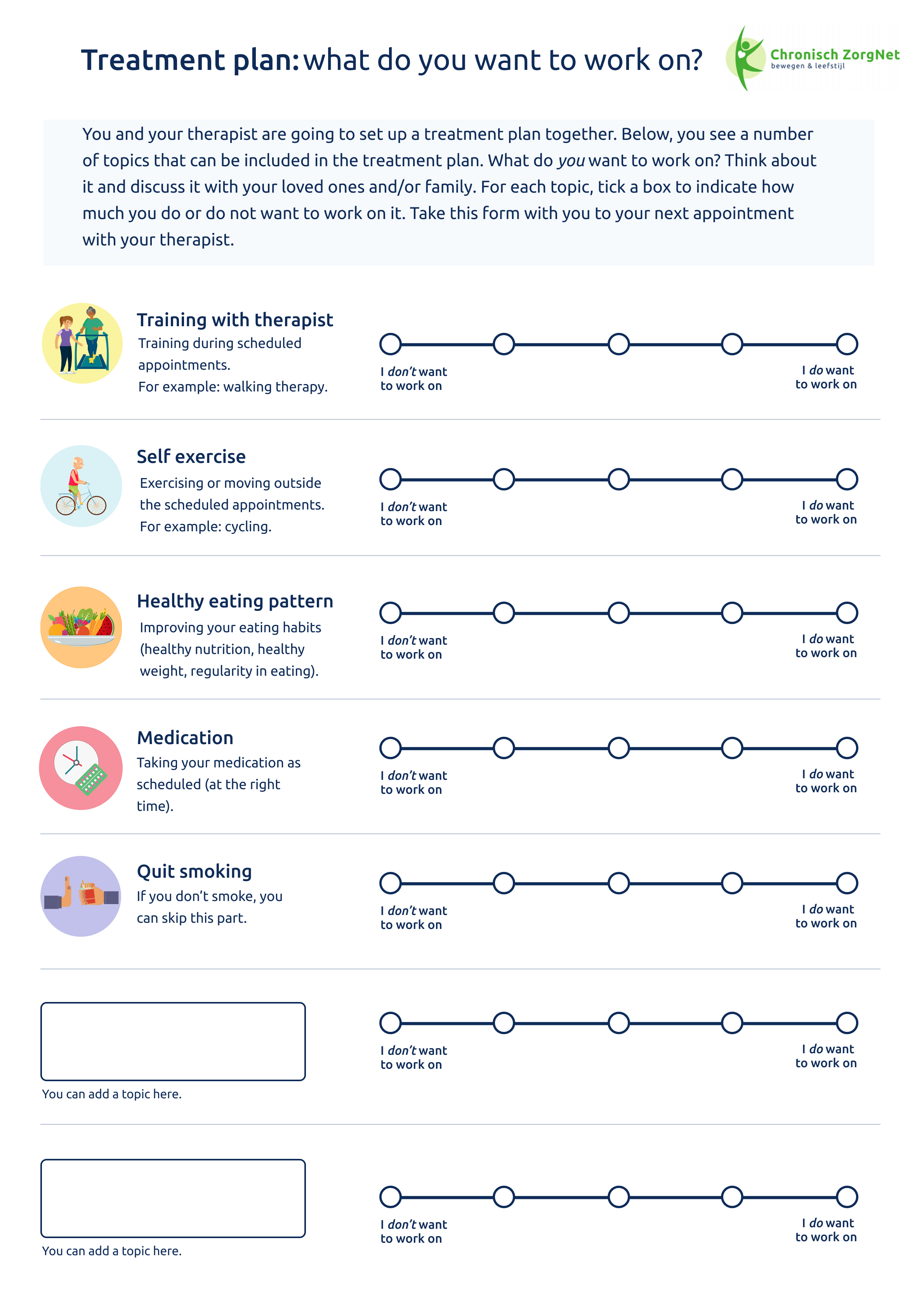

Supplement: Supplemental Material [file IANN_A_2540022_SM5047.zip › suppl_data/Supplementary Figure 2.jpg]

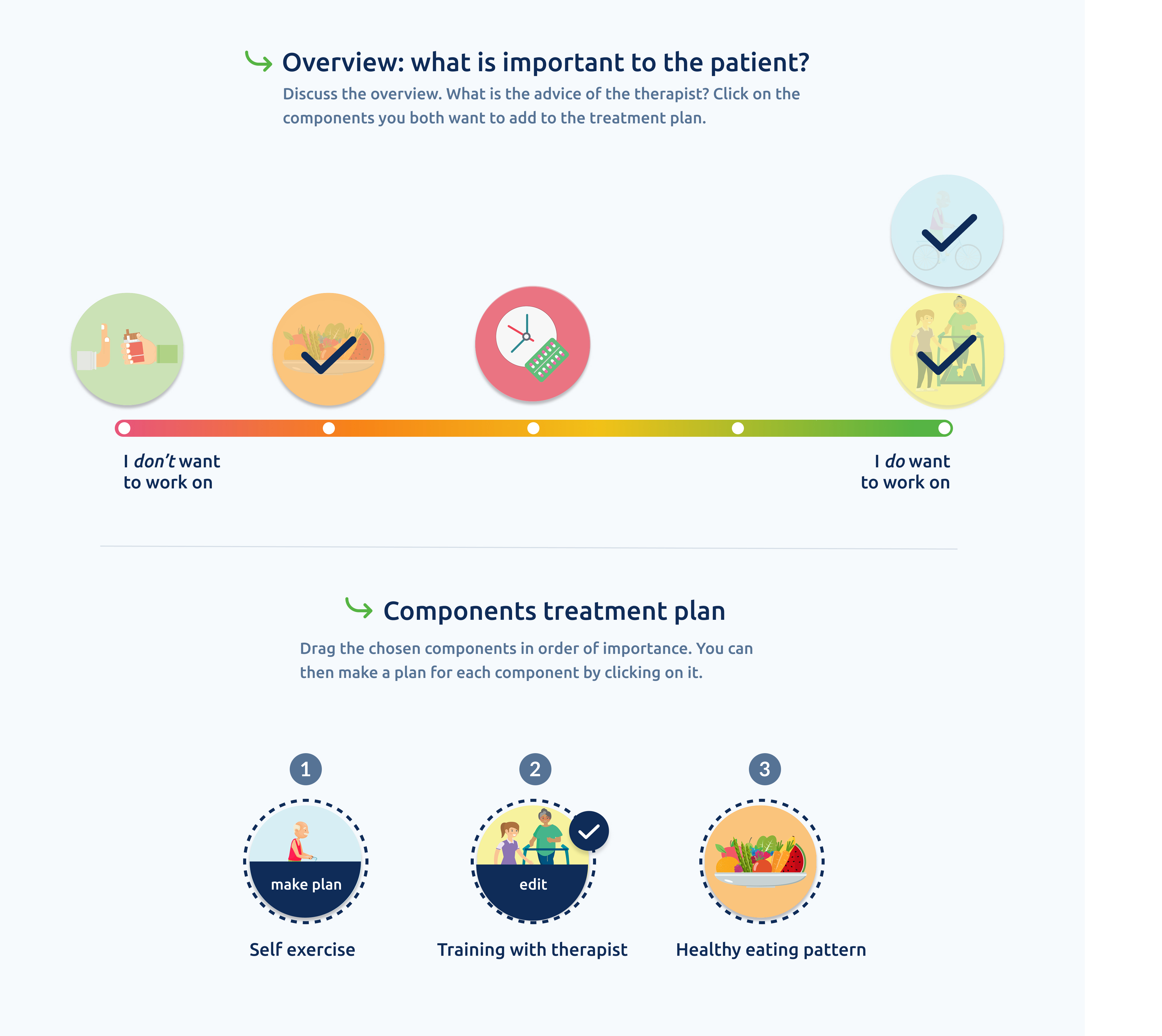

Supplement: Supplemental Material [file IANN_A_2540022_SM5047.zip › suppl_data/Supplementary Figure 3.jpg]

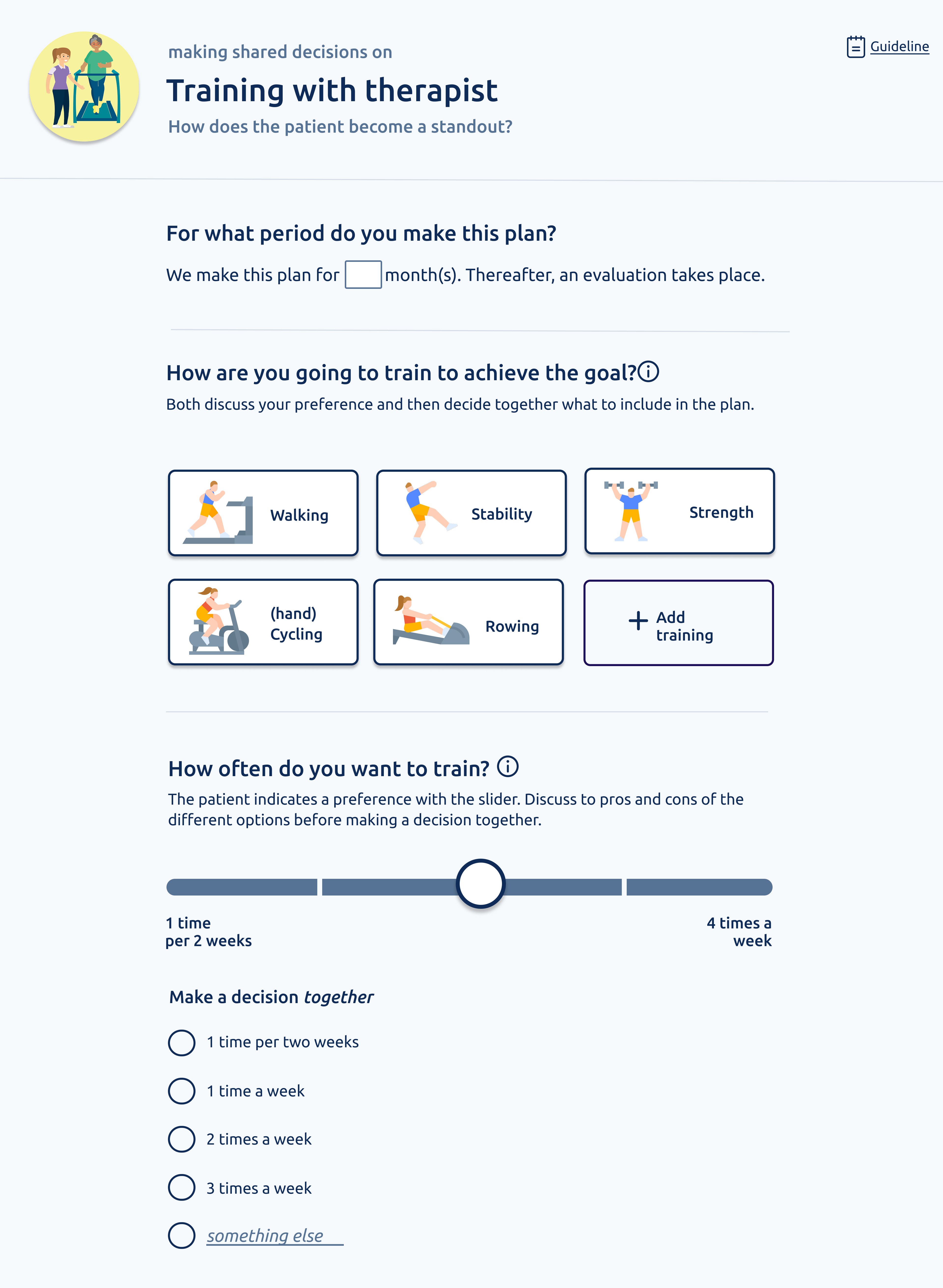

Supplement: Supplemental Material [file IANN_A_2540022_SM5047.zip › suppl_data/Supplementary Figure 4.jpg]

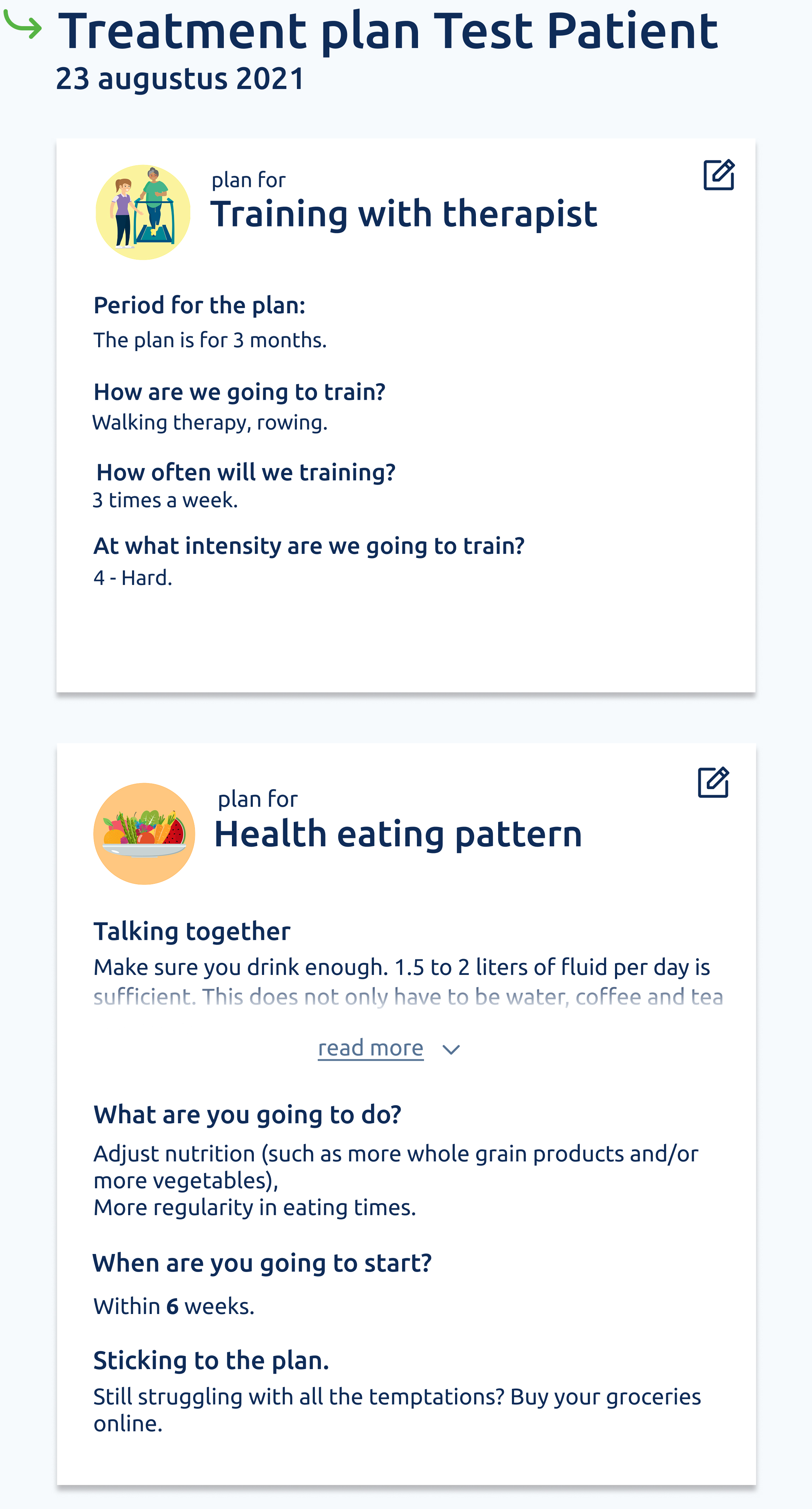

Supplement: Supplemental Material [file IANN_A_2540022_SM5047.zip › suppl_data/Supplementary Figure 5.jpg]

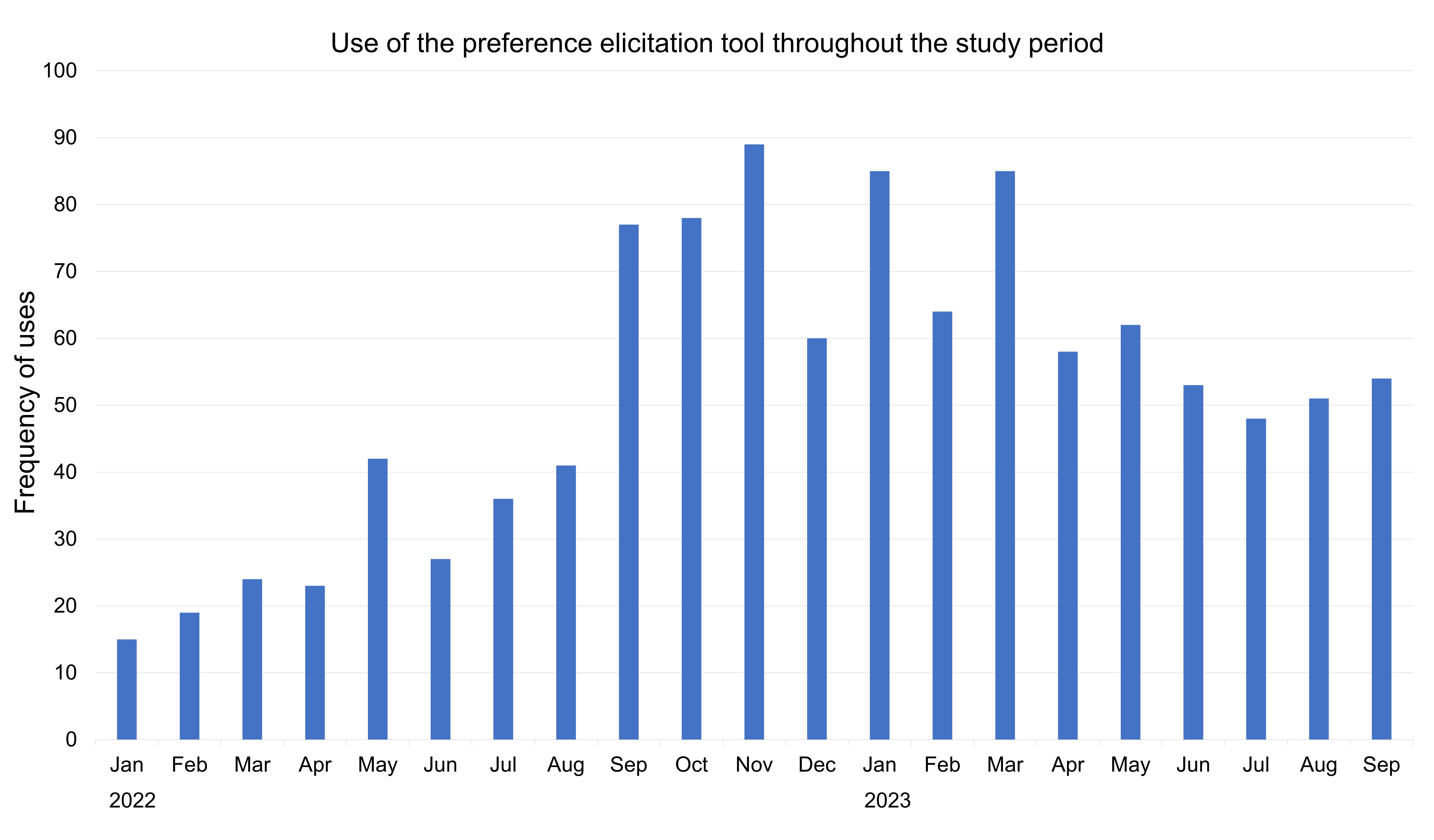

Supplement: Supplemental Material [file IANN_A_2540022_SM5047.zip › suppl_data/Supplementary Figure 6.jpg]
